# Individuals with High Metacognitive Ability Are Better at Divergent and Convergent Thinking

**DOI:** 10.3390/jintelligence11080162

**Published:** 2023-08-12

**Authors:** Lan Jiang, Chunliang Yang, Zhongling Pi, Yangping Li, Shaohang Liu, Xinfa Yi

**Affiliations:** 1 Key Laboratory of Modern Teaching Technology (Ministry of Education), Shaanxi Normal University, No. 199 Chang’an Road, Yanta District, Xi’an 710062, China; jianglan@snnu.edu.cn (L.J.); pizl@snnu.edu.cn (Z.P.); 2 Institute of Developmental Psychology, Faculty of Psychology, Beijing Normal University, 19 Xinjiekouwai Street, Haidian District, Beijing 100875, China; chunliang.yang@bnu.edu.cn (C.Y.); shaohang_liu@bnu.edu.cn (S.L.); 3 School of Foreign Studies, Xi’an Jiaotong University, No. 28 Xianning West Road, Xi’an 710049, China; yangpingli@snnu.edu.cn; 4 The Branch Center of National Collaborative Innovation Center of Assessment toward Basic Education Quality at Beijing Normal University, Shaanxi Normal University, No. 199 Chang’an Road, Yanta District, Xi’an 710062, China

**Keywords:** metacognitive ability, divergent thinking, convergent thinking, eye tracking

## Abstract

Is metacognitive ability a predictor of creative performance? Previous studies have produced conflicting findings. To clarify whether this relationship exists, the current study used eye tracking techniques and vocal thinking reports to explore creativity differences in individuals with different levels of metacognitive ability. One hundred and twelve participants completed the Metacognitive Ability scale, and were divided into two groups (with thirty participants in each group) based on their metacognition scores (the highest and lowest 27% of metacognitive ability scores). Then, participants in both groups completed two creative thinking tasks (AUT and CCRAT) while their eye behaviors were recorded by eye tracking. The results showed that participants with high metacognitive ability were better at divergent thinking, as evidenced by greater fixation and saccade counts, as well as smaller saccade amplitudes in the AUT task. In addition, Bayesian analyses provide anecdotal evidence that participants with high metacognitive ability tended to be better at convergent thinking. Furthermore, eye tracking results demonstrated that they exhibited longer fixation duration and more fixation count on the materials in the CCRAT task. These findings reflect an important role of metacognition in creative thinking, especially in divergent thinking.

## 1. Introduction

The rise of artificial intelligence, such as ChatGPT, has once again emphasized the importance of creative talent. Metacognition involves understanding how to learn and how to create, making it a crucial aspect of cultivating creative talent. Similar to the psychological mechanisms underlying other cognitive processes, metacognition monitors and regulates creative thinking (Sternberg and Lubart 1998). Multiple studies confirmed that individuals with varying metacognitive abilities exhibit different levels of creativity (Mevarech and Paz-Baruch 2022; Saggar et al. 2021; Urban et al. 2021).

Creative thinking is a complex process involving various cognitive skills, such as generating ideas, recognizing patterns, analyzing information, and evaluating alternatives. Metacognition plays a critical role in supervising and regulating these cognitive processes by monitoring, controlling, and modifying thinking strategies (Amabile 1983). Previous studies established that metacognitive ability intimately positively predicts creative achievements in different fields (Kaufman 2013). For instance, previous studies established that individuals with high metacognitive ability tend to be more flexible, adaptive, and open to new information, which are essential qualities for generating creative ideas (Bloom and Dole 2018; Kaufman and Beghetto 2013; Marino et al. 2018). Moreover, metacognitive monitoring allows individuals to be conscious of their cognitive processes, and to identify when certain thinking strategies are not working and need to be changed. However, some other studies have documented conflicting results regarding the relationship between metacognition and creativity (Fayena-Tawil et al. 2011; Mevarech and Paz-Baruch 2022). Some researchers argue that the monitoring function of metacognition may interfere with the spontaneity and originality of creative thinking (Preiss 2022; Zepeda and Nokes-Malach 2023). According to this view, self-awareness and monitoring may lead to self-censorship and constrain individuals’ creative potential.

Although various studies have confirmed the impact of metacognition on creative thinking, most of these studies employed relatively static psychometric methods. As a result, no studies have yet utilized dynamic and procedural experimental methods to investigate the impact of metacognition on the creative process. This could be a primary reason for the conflicting and scattered results among existing studies. Going beyond previous research, the current study aims to use eye tracking techniques and employ thinking-out-loud protocols to conduct a more comprehensive and dynamic exploration of individual creative processes from the perspective of task situations. This will help to further explore the differences in creativity performance and internal processes among individuals with different levels of metacognitive ability. The observed findings could serve as a basis for educators to develop individualized creativity training techniques tailored to individuals with different levels of metacognitive ability.

## 2. Literature Review

### 2.1. Metacognition

The essence of metacognition is “cognition of cognition” (Flavell 1979). Cognition mainly comprises knowledge stored by oneself and strategies used to solve problems, while metacognition involves monitoring, understanding, and adjusting one’s own knowledge and strategies. This helps individuals not only know what is important but also comprehend when, where, and how to appropriately apply various kinds of knowledge and strategies (Csikos 2022; Williams et al. 2016). In cognitive psychology, metacognition plays the role of “executive control”, which supervises thoughts, knowledge, and behavior. This kind of monitoring is achieved through introspection, knowledge of oneself as a cognitive subject, and adjustment of one’s way of thinking (Veenman and Spaans 2005). In short, metacognition describes people’s understanding and control of their thinking, which is a cognitive process that takes their own psychological state, cognitive process, and thinking mode as cognitive objects, and external manifestations are regulation and monitoring of cognitive activities (Efklides 2002; Flavell 1979).

The key components of metacognition are planning before tasks, monitoring during tasks, and evaluation during or after tasks (Efklides et al. 1999). Planning involves the “how” of executing activities, which includes three components: resource allocation, problem decomposition, and strategic planning (Moshman 2018). Resource allocation refers to how resources, such as time allocation, are distributed before the execution of activities to activate relevant knowledge. Problem decomposition involves breaking down a problem into several sub-problems or sub-goals to be solved separately and sequentially, including setting clear objectives and predicting outcomes. Strategic planning entails determining the order of achieving sub-goals in order to choose the most effective strategies, including setting priorities and predicting potential challenges (Efklides 2008). The plan focuses on the implementation aspects and delineates the steps involved in executing activities effectively (Veenman and Spaans 2005). It emphasizes the allocation of resources to optimize knowledge utilization, breaking down complex problems into manageable components, and strategically planning the order of completing sub-goals based on their significance and anticipated difficulties (Guo 2022). This systematic approach aims to enhance the efficiency and effectiveness of the overall execution process.

Monitoring and control refer to the supervision and coordination of cognitive processes (Rivers 2020). Monitoring requires the ability to assess one’s cognitive processes successfully, while control means using these assessments to modify behavior, such as coordinating time, allocating attention, adjusting strategies, tracking progress, and regulating emotions (Dinsmore et al. 2008). Monitoring and control play a crucial role in regulating cognitive processes. The ability to accurately monitor one’s cognitive performance and adjust behavior accordingly is vital for effective learning and problem solving (Paulewicz et al. 2020).

Various studies emphasize the importance of self-monitoring in promoting students’ metacognitive regulation and overall academic success. It is worth noting that high-achieving students demonstrate the adaptive application of knowledge and make wise choices regarding learning strategies (Paulewicz et al. 2020). This is because metacognitive monitoring utilizes metacognitive knowledge to help individuals formulate strategies to achieve cognitive goals (Medina et al. 2017). In recent years, many studies have highlighted the significant role of self-monitoring in areas such as students’ selection of learning strategies and information processing (Rivers 2020). Specifically, students with high academic performance exhibit flexibility in knowledge transformation and the selection of appropriate learning strategies (Grasset et al. 2022). Guo (2022) conducted a meta-analysis to examine the impact of metacognitive prompts (guiding students to adopt monitoring strategies) on self-regulated learning (SRL) and learning outcomes in a computer-based learning environment (CBLE) (Guo 2022). The results demonstrated that metacognitive prompts significantly enhanced self-regulated learning activities and learning outcomes.

Evaluation involves assessing the effectiveness of strategies, which includes the evaluation process, achievement, and the value and applicability of learned knowledge (Schraw and Dennison 1994; Schraw et al. 2014). This system of self-reflection allows individuals to monitor and adjust their thinking. It involves processes such as monitoring cognitive processes, assessing knowledge, controlling thinking, detecting errors, and using strategic thinking (Guo 2021). Through metacognition, individuals become aware of their thoughts, evaluate their understanding, regulate their thinking, identify and correct errors, and utilize effective strategies (Flavell 1979; Moshman 2018).

In recent years, there has been an increased use of eye tracking devices to record and identify the allocation of gaze time during cognitive monitoring (Karatekin 2007; Alemdag and Cagiltay 2018). Eye gaze is regarded as a reliable indicator of the focal point of active information processing, offering the advantage of being an accepted measure of implicit abilities and performance (Karatekin 2007). For example, compared to eye saccades, the distribution of gaze time reflects top-down processing, which is controlled by an individual’s ongoing cognitive processes (Karatekin 2007; Tsai et al. 2019). Roderer and Roebers (2014) used eye tracking technology to explore implicit monitoring processes in children (Roderer and Roebers 2014). Their study identified gaze position and duration as indicators of sustained information processing, and the results demonstrated the usefulness of eye tracking techniques in uncovering implicit and retrieval-limited monitoring processes. Hence, our objective is to gain intriguing insights into monitoring processes by applying eye tracking methods in creative thinking tasks.

### 2.2. Creative Thinking

Creative thinking is a high-level psychological activity involved in the creative process (Hong and Milgram 2010; Redifer et al. 2021). The term “creative thinking” was first introduced by Wallace and subdivided by Guilford into divergent thinking and convergent thinking, wherein each plays a different role (Guilford 1967; Lubart 2016). Different cognitive models describe creative thinking as a constant interplay between these two types of thinking (Cristofori et al. 2018; De Rooij and Vromans 2020; Fink et al. 2012). In various stages of creative problem solving, divergent and convergent thinking play different roles. They work in unison instead of separately (Runco 1992). In the creative process, individuals employ divergent thinking to generate ideas and then employ convergent thinking to identify criteria and limitations for evaluating and selecting ideas (Lubart 2016; Runco et al. 2023; Simonton 2015).

Divergent thinking, also referred to as “divergent thought”, is a cognitive process characterized by the generation of a multitude of alternative solutions or perspectives during problem solving or task completion. It entails the uninhibited production of numerous ideas, free from judgment or constraints, encompassing the exploration of non-traditional viewpoints and liberation from conventional or linear approaches (Yang et al. 2022). In the early stages, divergent thinking accentuates quantity as opposed to quality, as a broad range of ideas lays the foundation for a diverse repertoire of potential solutions (Runco et al. 2023). The fluidity and flexibility inherent in this mode of thinking enable individuals to explore multiple possibilities without being confined to a singular methodology (Benedek et al. 2016; Hao et al. 2017). Divergent thinking plays a critical role in cultivating creativity and novelty. By deviating from traditional modes of thinking, individuals are more likely to generate innovative and distinctive ideas that surpass the limitations of conventional problem solving approaches (Beaty and Silvia 2012; Silvia and Beaty 2012). To summarize, divergent thinking facilitates the exploration of alternative viewpoints, associations, and connections, thereby fostering original and creative solutions. It enables individuals to transcend orthodox and linear thinking patterns, stimulating the generation of a broad spectrum of ideas that contribute to novel and unique problem solving endeavors.

Divergent thinking plays a pivotal role in the generation of multiple alternative solutions, while convergent thinking is crucial for effectively resolving specific types of problems (Lubart 2016; Patston et al. 2021). Convergent thinking, also referred to as “convergence thinking” or “consensus thinking” is characterized by the pursuit of a singular correct solution, emphasizing accuracy, directionality, and closure. Convergent thinking leverages one’s existing knowledge stored in memory to facilitate a more focused cognitive approach towards a potential solution (Sriraman and Dickman 2017). In order to generate innovative and adaptive ideas for problem solving, a combination of divergent thinking for idea generation and convergent thinking for idea selection is typically employed (Cropley 2006). Indeed, both divergent and convergent thinking are indispensable in the creative process, whereby individuals strive to not only generate a wealth of novel ideas, perspectives, or problem solving approaches, but also integrate, evaluate, and identify the most optimal solutions within the given context.

In recent years, the field of eye tracking has emerged as a valuable tool for gaining deeper insights into human cognitive processing. Extensive research has demonstrated the association between eye movements and cognitive processes (Benedek et al. 2017; Ceh et al. 2021; Maheshwari et al. 2022b; Walcher et al. 2017). Studies have indicated that eye movements can serve as indicators of an individual’s thinking processes. Particularly in problem solving scenarios, analyzing an individual’s eye movement patterns can facilitate an understanding of the potential solutions they may generate. Maheshwari et al. (2022a) conducted a study that further confirmed the relationship between thinking patterns and eye movements (Maheshwari et al. 2022a). They compared the attention patterns of individuals performing the Alternative Uses Task (AUT) and the Remote Associates Task (RAT), and observed that individuals exhibited significantly longer average fixation durations on task materials during divergent tasks compared to convergent thinking. This finding suggests that individuals allocate more time to attending to experimental materials in order to generate a greater quantity of ideas. Similarly, Walcher et al. (2017) found a higher rate of fixations in conditions involving divergent thinking compared to those involving convergent thinking (Walcher et al. 2017). Another empirical study discovered a recombination of higher fixation rates and scan rates that corresponded to participants’ actual observations of objects during cognitive manipulations, indicating a potential coupling between eye activity and internal cognitive processes (Benedek et al. 2017). While most research on eye tracking and problem solving has focused on graph-based problems, where eye tracking is employed to investigate participants’ problem solving strategies, it remains crucial to explore the role of eye movements in tasks unrelated to graphs (Grant and Spivey 2003; Jankowska et al. 2018). More comprehensive research is warranted to gain a thorough and comprehensive understanding of the role of eye movements in effectively solving non-graph-based problems.

### 2.3. Potential Role of Metacognition in Creative Thinking

The impact of metacognition on creative thinking has long been a topic of interest for educators and psychologists. Supporting students in developing innovative thinking skills through metacognitive strategies has also been a focus of educational research. Some scholars propose that creative thinking is a metacognitive process model that is monitored and regulated by metacognition (Beaty and Silvia 2013; Feldhusen 1995; Mokhtari and Reichard 2002; Pesut 1990). Puryear (2015) assumed that metacognition plays an important role in creative thinking by acting on the thinking process (Puryear 2015). According to sequential effects, later ideas are typically more creative than earlier ones (Beaty et al. 2014a, 2014b), because the initial phase of divergent thinking requires flexible thinking to discover potentially valuable information from memory, whereas the later stage necessitates continuous evaluation, revision, and improvement of potential solutions (Avitia and Kaufman 2014; Sidi et al. 2020). In this process, individuals consciously reflect on and monitor their own thinking processes and strategy choices through metacognition, enabling them to generate ideas more flexibly (Beaty and Silvia 2013). Metacognition allows individuals to adjust their thinking methods and explore different perspectives and problem solving approaches when generating ideas. Individuals can self-assess and scrutinize their generated ideas, leading to revision and improvement. This process helps individuals identify and correct potential errors or shortcomings, thereby enhancing the quality and effectiveness of solutions (Sidi et al. 2020). This requires the use of both top-down control processing and bottom-up associative processing to generate more creative ideas (De Rooij and Vromans 2020; Liu et al. 2023). Bottom-up spontaneity is insufficient for divergent thinking, as the process also requires monitored retrieval to select appropriate concepts while suppressing irrational connections. Monitored retrieval and idea selection, along with the suppression of irrational associations, play a crucial role in cognitive processes and creative thinking (Liu et al. 2023). These cognitive mechanisms enable individuals to refine and enhance the quality of their ideas by actively monitoring and controlling the retrieval of relevant information from their memory and by selectively choosing the most appropriate ideas (Saggar et al. 2021).

In addition, cognitive flexibility positively predicts creative problem solving performance (Takeuchi et al. 2010; Zabelina and Robinson 2010). Cognitive flexibility is the ability to adapt and switch between cognitive processes, strategies, or perspectives (Martin et al. 2011). It allows individuals to approach problems from multiple angles, explore alternative perspectives, and generate creative ideas. This is achieved through shifting attention, breaking mental set, integrating perspectives, and overcoming functional fixedness (Diamond 2013). Cognitive flexibility breaks rigid cognitive patterns, embraces fresh perspectives, integrates diverse viewpoints, and enables innovative problem solving by thinking beyond predefined functions. Overall, cognitive flexibility enhances adaptability and creativity in approaching various challenges (Cristofori et al. 2018). Cognitive stability can also predict analytical thinking performance (Razumnikova 2007), mainly by increasing working memory capacity and consolidating task information to promote effective analysis (Nijstad and Stroebe 2006).

Although researchers have investigated the relationship between metacognition and creativity from various theoretical perspectives, few empirical studies have explored and verified it from a dynamic procedural perspective. Empirical research showed that excellent creators are better at intentionally participating in the metacognitive process (Liu et al. 2023). This includes planning and managing their time before tasks, monitoring their cognitive processes while performing the tasks, and evaluating their thinking state to adjust strategies in a timely manner for better problem solving (Zimmerman 1998). Intentional monitoring promotes creative performance. Conversely, better creators are skilled at consciously using metacognition to monitor their cognition (Akin et al. 2007; Urban et al. 2021). Studies have also found that artists are better at monitoring the painting process (Fayena-Tawil et al. 2011; Kay 1991). For example, previous studies asked students who had no design experience to design logos for drinks. The product designers reported stronger improvements from initial sketches to final sketches (Jaarsveld and Leeuwen 2005). Similarly, Wetzstein and Hacker (2004) found that participants who received prompts encouraging self-reflection made greater progress in designing drawings and solutions compared to those who did not receive prompts (Hong et al. 2016; Wetzstein and Hacker 2004). Jia and colleagues (2019) conducted a review of previous research on the role of metacognition in creative thinking (Jia et al. 2019). The review found that although an increasing number of studies suggest the potential importance of metacognition in creative thinking, there is still a lack of consensus among empirical research findings; for example, in a study conducted by Preiss et al. (2016) with university and vocational Chilean students as participants (Preiss et al. 2016), it was found that metacognition did not significantly predict creative performance. The review emphasizes the need for future studies to explore the underlying mechanisms through which metacognition influences creative thinking.

The core of this “synchronous linkage” changing relationship lies in the fact that creative strategies guide thinking and encourage individuals to imagine or invent new products by combining, changing, or applying existing concepts (Bai et al. 2021). Specifically, in a creative thinking task, individuals must focus their attention on the creative problem and the strategies they know may contribute to solving the problem, as well as evaluate the quality of the solution or creative idea that they generate. These complex processes require the use of working memory. This is because working memory is an executive system responsible for an individual’s ability to plan and monitor the encoding and storage of information, guiding individuals to shift their focus of attention (Baddeley et al. 1998). Secondly, it relies on the conscious or unconscious control of metacognition over these thoughts, attention, memories and actions (Hargrove and Nietfeld 2015). Metacognition plays a crucial role in managing cognitive processes involved in creative thinking. It contributes to the generation, evaluation, and selection of ideas, understanding thinking strategies, assisting individuals in regulating attention, refocusing on pertinent aspects, and avoiding interference or biases (Veenman and Spaans 2005). It organizes and utilizes existing knowledge and memories, integrating them with novel ideas. Metacognition also aids in promoting planning, monitoring, and adjusting behaviors, fostering flexibility and an experimental mindset (Koriat and Levy-Sadot 2000).

Eye tracking technology is considered an effective tool for measuring an individual’s attention process and pattern, providing strong support for exploring an individual’s cognitive dynamics (Pi et al. 2018). For instance, previous studies have confirmed that the longer an individual looks at material information, the more attention he or she pays to that information (Michinov et al. 2015).

### 2.4. The Present Study

Previous studies have investigated the relationship between metacognition and creative thinking, but the results have been inconclusive. The observed disparity in findings across studies conducted in different countries can be attributed to variations in participant demographics. These distinctions may arise from factors such as regional cultural influences or specific knowledge backgrounds, as well as variances in age distribution within the participant samples. Additionally, inconsistent research outcomes may be attributable to disparate implementation of assessment tools. Previous investigations into creativity have encompassed diverse task scenarios, including tasks such as visual and verbal creativity.

Furthermore, limitations within a considerable body of research lie in its emphasis on investigating the static psychological relationship between metacognition and creative thinking primarily at the behavioral level. This perspective fails to provide a comprehensive understanding of the role played by cognitive processes, such as attention and memory, in the creative endeavor, particularly among individuals with varying levels of metacognitive abilities. To address these limitations, the present study aims to explore the impact of individual metacognitive capacities and contextual attention on creative thinking. This investigation will involve a sample of college students from a specific Chinese university and will employ behavioral tasks alongside eye tracking technology.

Summarily, plausible explanations for the absence of consensus in prior studies include disparities in participant demographics, variations in assessment tools and task scenarios, as well as an inclination towards investigating static psychological associations rather than dynamic cognitive processes inherent in creativity. The present research endeavors to overcome some limitations by examining the relationship between metacognitive ability and creative thinking in individuals using dynamic and procedural measures, within a targeted sample of Chinese university students, employing both behavioral measures and eye tracking techniques.

The findings on metacognitive functioning and contextual attention in relation to creative thinking provide guidelines for educators and researchers to promote creativity in individuals with different metacognitive abilities. Educators can tailor instruction and provide targeted support to enhance creative thinking. Metacognitive training programs can be developed to teach students how to regulate attention and avoid biases. Assessments can evaluate metacognitive strengths and inform feedback. Researchers can use these findings to design experiments and deepen the understanding of the relationship between metacognition, contextual attention, and creativity. Collaboration between disciplines can lead to innovative strategies and interventions. Overall, these findings have practical implications for fostering creativity in education and advancing research in the field.

## 3. Method

### 3.1. Participants

In total, 112 college students (95 females, 17 males, *M* = 21.91, *SD* = 1.807) were randomly recruited online from a university in China. The distribution of participants across disciplines is as follows: humanities account for 46.64%, natural sciences account for 44.39%, engineering disciplines account for 6.73%, and arts disciplines account for 2.24% (including music, sports, and visual arts). All participants completed “The Metacognitive Ability Scale for College Students” (Sun and Tian 2015). Of these, we selected 30 participants (28 females, 2 males, *M* = 19.333, *SD* = 1.061) with the highest 27% of metacognitive ability scores and assigned them to the high metacognitive ability group. The 30 participants (26 females, 4 males, *M* = 19.533, *SD* = 1.196) with the lowest 27% of metacognitive ability scores were assigned to the low metacognitive ability group. Then, we removed the middle portion of participants. The two groups did not differ in age and gender, but there was a significant difference in metacognitive ability between the two groups, *t* (58) = 13.762, *p* < .001, *d* = 3.55. A total of 60 participants completed the follow-up eye movement experiment.

As no previous studies have employed the same materials and experimental design as those in the current study, we hence did not conduct *a priori* sample size justification. Instead, we performed post hoc sensitivity analyses to check the statistical power of the current sample size. For the critical effects explored in the present study (Fluency: Cohen’s *d* = 0.88; Flexibility: *d* = 0.89; Originality: *d* = 0.81), the calculated statistical power for these comparisons has all surpassed .80 with a sample size of 60.

The visual acuity of all participants met the requirements of eye movement experiment. Each participant received RMB 30 as task compensation. All participants signed a consent form to participate. The current study was proved by the Ethics Committee of Key Laboratory of Modern Teaching Technology (Ministry of Education), Shaanxi Normal University.

### 3.2. Materials

#### 3.2.1. Alternative Uses Task (AUT)

The AUT was employed to measure participants’ divergent thinking ability (Guilford 1967; Runco 1992). In this test, participants were presented with three items, including “brick”, “newspaper”, and “pen”. Their task was to come up with as many novel and unique uses for each item as possible during three minutes. For each participant, the three items were presented in a random order.

Two raters engaged in joint discussion and consolidated synonymous or similar viewpoints in participants’ responses (merging duplicate or synonymous points). Next, they evaluated the three dimensions of divergent thinking, including fluency (i.e., the number of valid responses, one point for a valid answer), flexibility (i.e., the number of categories represented by the responses, count as many categories as there are), and originality (i.e., the uniqueness and rarity of the responses) (McCarthy et al. 2018). The originality score for each response was determined by its frequency of occurrence among all responses provided by all participants (Plucker et al. 2014). Specifically, a response was awarded 2 points if it was unique and original, 1 point if it accounted for 2% to 4% of all responses, and 0 points for all other responses (van de Kamp et al. 2015).

Two doctoral candidates in psychology first collaborated to remove invalid answers and counted the number of valid answers as the fluency score. Then, they independently scored the flexibility and originality of the valid answers according to the aforementioned scoring criteria (Plucker et al. 2014). ICC was calculated to quantify consistency between raters, which showed that inter-rater correlations on flexibility were satisfactory (ICC = .90), and the same for originality (ICC = .94). These results indicate a high level of agreement between the two raters’ evaluations.

#### 3.2.2. Chinese Compound Remote Associates Test (CCRAT)

The CCRAT was utilized to measure participants’ convergent thinking ability (Wu et al. 2017). In each trial of the CCRAT, participants were presented with three simplified Chinese clue characters, such as “疗” (liao; treatment), “防” (fang; defense), and “统” (tong; completely). They were required to find a target Chinese character which could be combined with all three cue characters to form a meaningful two-character Chinese word. For example, the Chinese character “治” (zhi; ruling) could be combined with each of the cue characters to form the words “治疗” (zhi-liao; treatment), “防治” (fang-zhi; prevention), and “统治” (tong-zhi; ruling), respectively. Each correct answer scores one point, while incorrect answers do not score any points. A higher score indicates stronger convergent thinking ability. The level of integrative thinking ability is ultimately reflected by counting the number of correct answers in the CCRAT task (Hung and Wu 2021; Wu and Chen 2021; Zhu et al. 2019).

#### 3.2.3. Metacognitive Ability Scale for College Students

We adopted the “College Students’ Metacognitive Ability Scale” (Sun and Tian 2015). The scale measures four sub-dimensions of metacognitive ability. Metacognitive planning comprises seven items, primarily focusing on the abilities that college students possess before engaging in a cognitive task. It reflects their awareness of their cognitive abilities, cognitive strategies, and knowledge related to cognitive tasks; for example, “I not only possess various learning strategies but also understand when, where, and why to use them” (Cronbach’s α = .75). Metacognitive monitoring consists of six items and primarily assesses the abilities that college students possess during the execution of a cognitive task. It reflects their capacity to recognize tasks, monitor and evaluate their cognitive progress, and predict the outcomes of their progress; for instance, “During the learning process, I ask myself if I have achieved my learning objectives” (Cronbach’s α = .77). Metacognitive adjustment comprises six items and involves the abilities that college students have while executing a metacognitive monitoring task. It reflects their capabilities in organizing their resources, determining the specific steps required to complete a task, adjusting the speed and intensity of their work, and sequencing their activities; for example, “To adapt to different conditions or requirements, I try to change my learning methods” (Cronbach’s α = .72). Finally, metacognitive evaluation includes five items, primarily assessing the abilities that college students possess after completing a cognitive task. It reflects their capability to assess their cognitive efficiency and effectiveness, and proactively stimulate their motivation for future learning by reflecting on their experiences; for example, “After completing a task, I ask myself if there is an easier way” (Cronbach’s α = .70). Metacognitive ability was indexed as the sum of the scores from these four dimensions. The scale has demonstrated excellent reliability and validity with Cronbach’s α at .90 and KMO at .85.

### 3.3. Apparatus

We used an Eyelink 1000 eye tracker from SR Research Ltd. In Mississauga, Ontario, Canada, with a sampling rate at 1000 Hz. The experimental materials were presented on a 19-inch display screen, and the distance between the screen and the participants was 60 cm. As the Eyelink 1000 system only records eye movements in one eye, participants had both eyes fixed on the screen, but only the movements of the right eye were captured. If the eye tracker could not detect the right eye, then the left eye was tracked instead.

### 3.4. Procedure

The study lasted approximately 40 mins. Initially, demographic information was collected from all participants. Next, the requirements for oral report were explained, and participants had the opportunity to practice the oral report. Afterward, they were directed to the eye tracking room for the formal experiment.

Before the formal experiment, a 9-point calibration and validation process was conducted to ensure accurate collection of eye movement data. Calibration of eye tracking at 9 points is an important step in the process of using an eye tracker, aiming to ensure the accuracy and reliability of eye tracking data (Ceh et al. 2021; Maheshwari et al. 2022b). Before calibration begins, the system displays a sequence of calibration points consisting of 9 fixed positions (usually squares or circles; squares were used in this study). The participants need to focus their gaze on each calibration point until the system indicates moving to the next point. Typically, each calibration point lasts a few seconds. After calibration, the eye tracker adjusts and calibrates its tracking algorithm and device settings based on the participant’s eye tracking data (Akbari et al. 2023; Benedek et al. 2017; Walcher et al. 2017). A 9-point calibration is necessary; firstly, calibration can correct any errors and biases that may exist in the eye tracker, thereby improving the accuracy and precision of the data. The calibration process utilizes the participant’s eye movements to establish accurate eye movement patterns and align them with the actual positions on the screen (Jankowska et al. 2018). Secondly, through calibration, the eye tracker learns individual differences of the participant, such as pupil size, corneal reflection, and other factors, to better adapt and track each participant’s eye movements. In addition, calibration also helps assess and eliminate potential environmental factors’ effects on eye tracking data, such as lighting conditions, monitor settings, and other factors (Zhang et al. 2020). Finally, accurate eye tracking data are crucial for understanding visual attention, cognitive processes, decision-making behaviors, and other aspects in experiments or research (Michinov et al. 2015). By performing eye tracking calibration, data reliability and comparability are enhanced, thereby increasing the accuracy and interpretability of experimental results. In summary, calibration of eye tracking at 9 points is a crucial step in eye tracking research as it ensures data accuracy and reliability, providing a solid foundation for effective eye tracking analysis.

Participants then completed divergent thinking tasks (AUT) and convergent thinking tasks (CCRAT), with AUT and CCRAT appearing in a random order in order to overcome the sequential effect, with their eye movement data being collected during each task. To avoid potential interference induced by eye fatigue, all participants performed the experimental tasks before 6 pm (Akbari et al. 2023).

During the AUT, participants were first informed that they needed to verbally report as many novel and unique uses as possible for a given presented item during three minutes. Once the instructions were understood, the formal experiment commenced. Each trial began with a fixed point “+” at the middle of the screen, followed by the presentation of a random-selected item (e.g., brick). The presentation of each item lasted three minutes, and participants could press the space bar to proceed to the next item once they had exhausted their ideas. It takes nine minutes to complete three random items (“brick”, “newspaper”, and “pen”).

The experimental procedure for CCRAT was similar to that of AUT, except that the presentation time for each item was set to 20 s. As CCRAT is a challenging task, two practice trials were included to familiarize participants with the task. Responses to practice trials were excluded from analyses. After the practice phase, the formal experiment began and the order of these items was randomized.

### 3.5. Data Analysis

In the study, SPSS was primarily used to compare the differences in behavior and eye tracking data between two groups of participants with different metacognitive levels. The behavioral data comprise answers to divergent thinking and convergent thinking tasks, mainly obtained through recording participants’ verbal reports during the tasks. Eye tracking data was collected using an eye tracking device, which includes measures such as fixation counts and other eye movement trajectories during the performance of behavioral tasks.

## 4. Results

### 4.1. AUT (Divergent Thinking)

**Figure 1:**
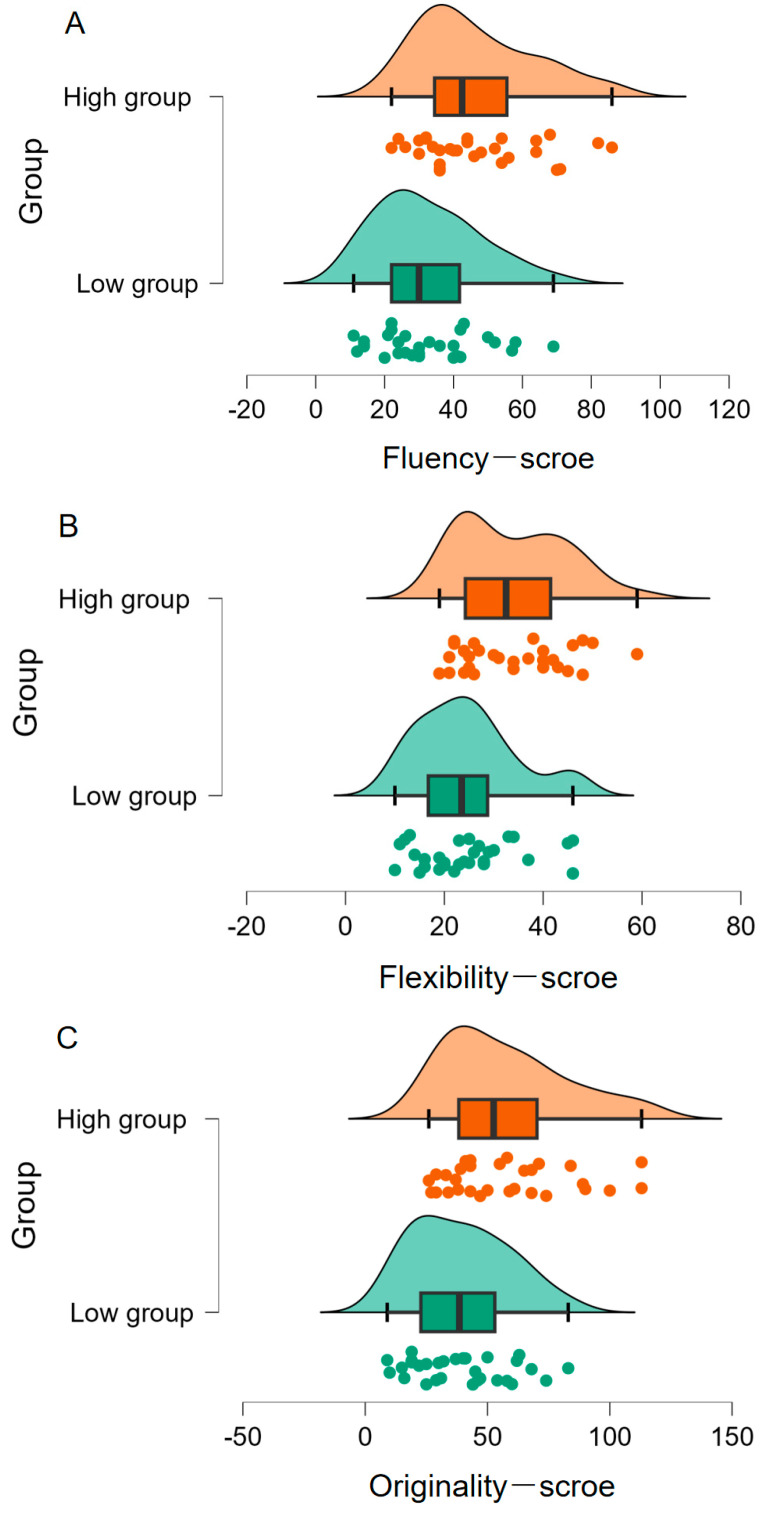
Differences in fluency (**A**), flexibility (**B**) and originality (**C**) scores of divergent thinking between two groups of high and low metacognitive ability.

As illustrated in Figure 1A, individuals with high metacognitive ability (*M* = 46.57, *SD* = 17.13) showed significantly higher levels of fluency compared to those with low metacognitive ability (*M* = 32.37, *SD* = 14.91), difference = 14.20 [22.50, 5.90], *t*(58) = 3.43, *p* = .001, *d* = 0.88, *BF*_10_ = 27.81. As shown in Figure 1B, participants with high metacognitive ability (*M* = 33.70, *SD* = 10.71) also demonstrated greater levels of flexibility than those with low metacognitive ability (*M* = 24.53, *SD* = 9.98), difference = 9.17 [14.52, 3.82], *t*(58) = 3.43, *p* = .001, *d* = 0.89, *BF*_10_ = 28.19. Additionally, individuals with high metacognitive ability (*M* = 57.57, *SD* = 25.18) showed higher levels of originality than those with low metacognitive ability (*M* = 39.10, *SD* = 19.88), difference = 18.47 [30.19, 6.74], *t*(58) = 3.15, *p* = .003, *d* = 0.81, *BF*_10_ = 14.18 (Figure 1C).

Overall, the above results reflect that individuals with high metacognitive ability are also good at divergent thinking, as they are able to generate creative ideas more fluently and flexibly, and their generated ideas are of greater originality.

**Figure 2:**
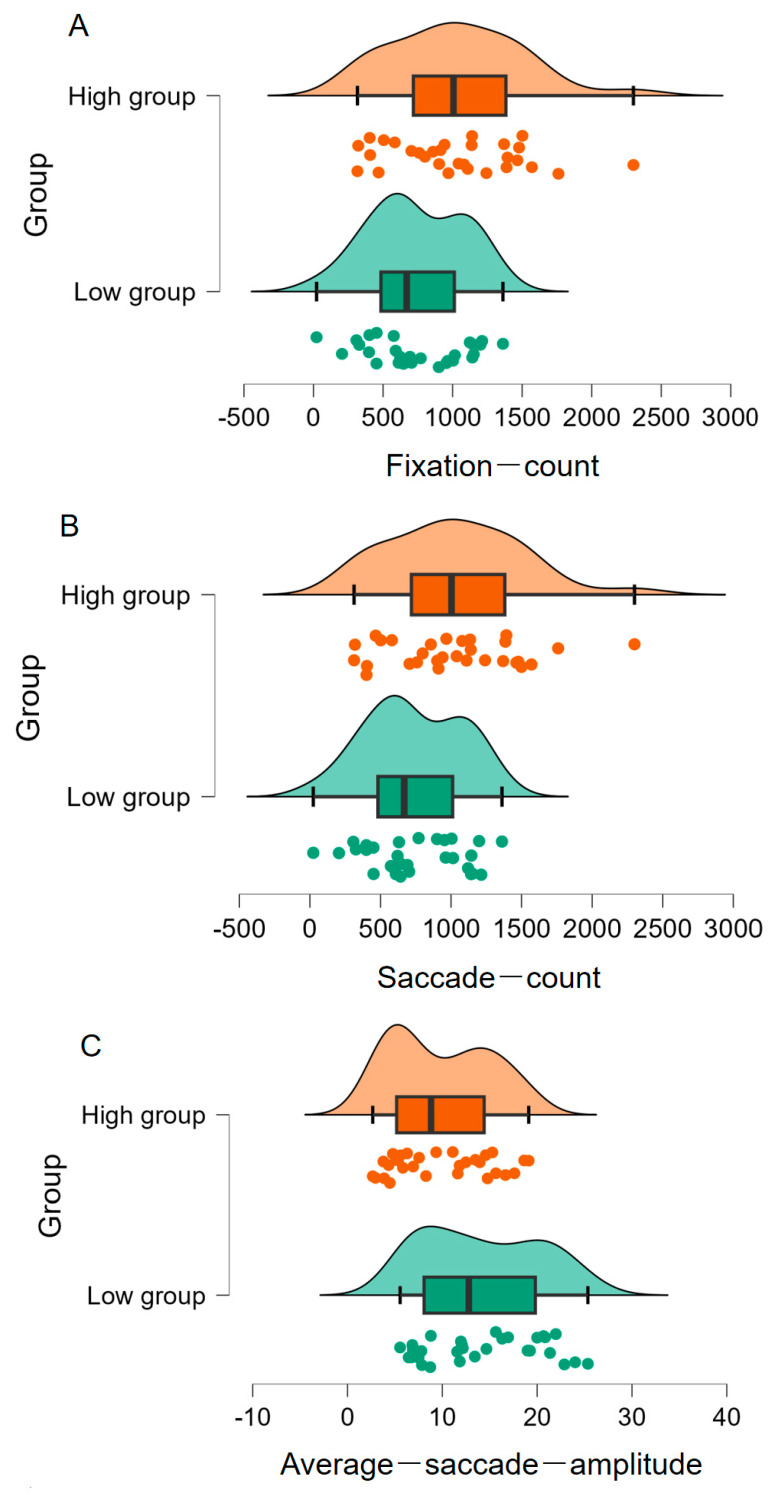
Differences in the three eye movement indicators (fixation count (**A**), saccade count (**B**), and average saccade amplitude (**C**)) in the AUT between two groups of high and low metacognitive ability.

Regarding the eye movement results, participants with high metacognitive ability (*M* = 1028.07, *SD* = 469.44) showed higher fixation counts than those with low metacognitive ability (*M* = 741.43, *SD* = 342.12), difference = 286.63 [498.92, 74.34], *t*(58) = 2.70, *p* = .009, *d* = 0.70, *BF*_10_ = 5.12 (refer to Figure 2A). Additionally, as illustrated in Figure 2B, individuals with high metacognitive ability (*M* = 1026.20, *SD* = 469.82) displayed more saccade counts than those with low metacognitive ability (*M* = 740.23, *SD* = 342.26), difference = 285.97 [498.40, 73.53], *t*(58) = 2.70, *p* = .009, *d* = 0.70, *BF*_10_ = 5.03. Moreover, participants with low metacognitive ability (*M* = 14.17, *SD* = 6.16) exhibited larger average saccade amplitude than those with high metacognitive ability (*M* = 9.80, *SD* = 5.21), difference = 4.37 [1.42, 7.32], *t*(58) = 2.97, *p* = .004, *d* = 0.766, *BF*_10_ = 9.17 (refer to Figure 2C).

These findings suggest that individuals with high metacognitive ability possess greater fixation counts, saccade counts, and smaller average saccade amplitude, reflecting that they focus more attention on experimental stimuli.

### 4.2. CCRAT (Convergent Thinking)

**Figure 3:**
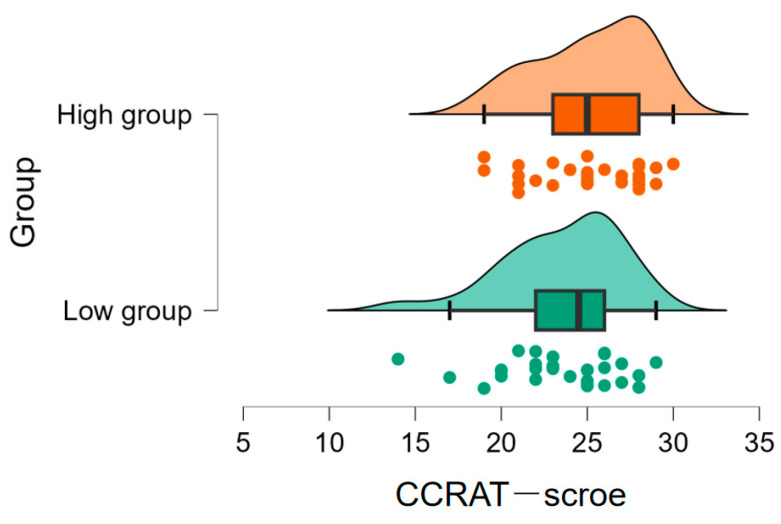
Differences in convergent thinking scores between two groups of high and low metacognitive ability.

**Figure 4:**
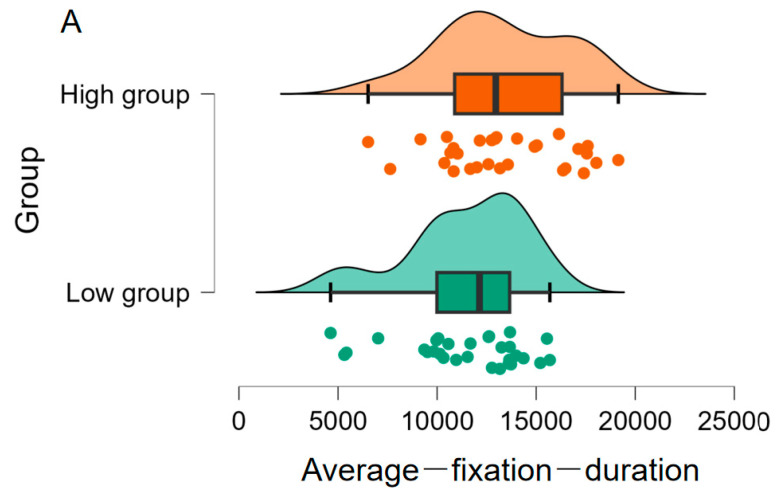

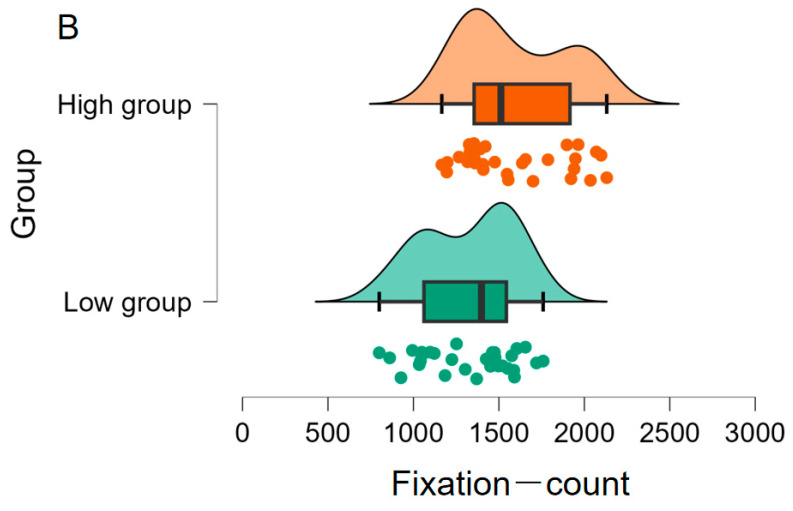
Differences in the two eye movement indicators (Average fixation duration (**A**), Fixation count (**B**)) in the CCRAT between two groups of high and low metacognitive ability.

As shown in Figure 3, the high (*M* = 23.53, *SD* = 3.44) and low (*M* = 25.20, *SD* = 3.17) metacognitive ability groups showed no significant difference in their convergent thinking performance based on the *p* value of a frequentist test, difference = 1.67 [−3.37, 0.04], *t*(58) = 1.95, *p* = .06, *d* = 0.50, but the Bayesian evidence (*BF*_10_ = 1.27) indicates a noteworthy tendency. Furthermore, participants with high metacognitive ability (M = 13373.88, SD = 3221.68) exhibited longer fixation duration than those with low metacognitive ability (M = 11461.76, SD = 2982.79), difference = 1912.12 [3516.67, 307.56], *t*(58) = 2.39, *p* = .02, *d* = 0.62, *BF*_10_ = 2.71 (Figure 4A). Additionally, participants with high metacognitive ability (M = 1595.47, SD = 307.99) showed greater fixation counts than those with low metacognitive ability (M = 1322.10, SD = 272.29), difference = 273.37 [423.61, 123.13], *t*(58) = 3.64, *p* = .001, *d* = 0.94, *BF*_10_ = 0.67 (Figure 4B). These findings indicated that participants with high metacognitive ability tended to be better at convergent thinking and paid more attention to important information while performing the CCRAT task.

## 5. Discussion

The current study utilized behavioral tasks and eye tracking techniques to investigate whether individuals with different levels of metacognitive ability possess different levels of creativity. The results revealed significant differences in both divergent thinking performance and eye movement patterns between the two groups. Specifically, participants with high metacognitive ability performed better in the three dimensions of divergent thinking (i.e., fluency, flexibility, and originality) and exhibited greater fixation and saccade counts, but smaller average saccade amplitude. In addition, Bayesian analyses provide anecdotal evidence that participants with high metacognitive ability tended to be better at convergent thinking. Furthermore, eye tracking results demonstrated that they exhibited longer fixation duration and more fixation count on the materials in the CCRAT task. These findings suggested that metacognition plays a positive role in creative thinking, especially in divergent thinking. Additionally, people with different levels of metacognitive ability exhibit different attention patterns when performing creative thinking tasks. Overall, these findings are particularly valuable in revealing the role of metacognition in creative thinking.

### 5.1. The Role of Metacognition in Divergent Thinking

Participants with high metacognitive ability showed better performance in divergent thinking compared to those with low metacognitive ability. This finding is consistent with those of previous empirical studies (Hargrove and Nietfeld 2015), which observed that individuals who are better at using metacognition produce better creative performance (Benedek et al. 2016). Notably, recent studies have found a positive correlation between metacognition and performance on complex creativity tasks, particularly in product improvement tasks, further supporting the idea that the more complex a creativity task is, the more metacognition is involved (Urban et al. 2021; Urban and Urban 2023). These findings are supported by sequential effects and top-down control theory, which are discussed in the Introduction section (De Rooij and Vromans 2020; Sidi et al. 2020; Sternberg 2017). Moreover, meta-analysis results showed that metacognitive training is effective in enhancing individuals’ creativity compared to other teaching methods (Perry et al. 2019). These findings jointly highlight the importance of using metacognition to plan creative activities, monitor, and evaluate the thinking process (Mevarech and Paz-Baruch 2022).

Going beyond previous research, the current study utilized eye movement techniques to record online eye movement patterns for the aim to explore people’s internal cognitive processes when solving creative problems. Eye movement indicators revealed that, in the AUT task, participants with high metacognitive ability exhibited greater fixation and saccade counts, as well as lower average saccade amplitude, compared to those with low metacognitive ability. Previous findings established that visual fixation positively relates to concept comprehension, and saccade amplitude is negatively associated with cognitive processing speed and typically reflects poor levels of material processing. These eye movement findings are consistent with those from previous studies. For instance, Knoblich et al. (2001) used the matchstick task to explore the impact of insight on problem solving and found that participants who successfully solved the problem paid more attention to key information during the problem solving process (Knoblich et al. 2001). This suggests that top-down processing plays a key role in guiding selective attention (Liu et al. 2023).

The observed findings support the controlled-attention theory, which asserts that creative ideas arise from top-down control over attention and cognition (Beaty et al. 2014a; Nusbaum and Silvia 2011). Superior metacognitive ability allows individuals to assess and evaluate the gap between their current cognitive state and their desired state, and successfully solve problems by adapting their cognitive strategies and attention patterns in a timely manner (Macgregor et al. 2001). The metacognitive process model understands metacognition as both bottom-up monitoring and top-down control. First-order cognition includes functions such as object recognition, decision making, coding, and characterization. At the meta level, these first-order cognitive functions are processed and modulated upward to enhance object-level functionality (Fleur et al. 2021).

### 5.2. The Role of Metacognition in Convergent Thinking

Two groups of students showed no significant difference in convergent thinking performance based on the *p* value of a frequentist test, but the Bayesian evidence (*BF*_10_ = 1.27) indicates a noteworthy tendency. This can be considered anecdotal evidence that participants in the high metacognitive ability group performed the convergent thinking task better. Furthermore, the differences in the attention patterns between the two groups were detected. Specifically, the high metacognitive ability group spent more time focusing on the cue words displayed on the screen during problem solving. A possible explanation is that solving a problem requires individuals to compare their answers with other potential response options, and metacognition is necessary to monitor attention and modulate strategies for comparing various stimulus characters to determine the correctness of a response. Individuals with high metacognitive ability are better at using top-down monitoring and retrieval processes to suppress unreasonable connections while selecting appropriate concepts from activated ones, thereby guiding initial concepts towards more reasonable target concepts from top to bottom. As a result, they tend to pay more attention to key information. This discovery supports findings from previous studies that used eye tracking technology to explore how novices and experts solve circuit problems. For instance, Rosengrant et al. (2021) documented that while both novices and experts had similar staring patterns for circuit components, experts looked back at the circuit during problem solving while novices did not (Rosengrant et al. 2021).

## 6. Limitations and Future Research Directions

Some advantages and limitations of the current study should be elaborated. The findings observed here reflect the value of metacognitive skills in promoting creative development, especially in terms of divergent thinking. The current study also provided promising results for the intervention of training metacognition to improve creativity. Furthermore, this study explored the role of metacognition in creativity from the perspective of attention patterns, providing new perspectives on mechanism exploration.

The current study, however, suffered from several limitations. First, although the current study explored the role of metacognition in creativity thinking by analyzing behavior and eye movement data, all observed findings are correlational. Future studies are encouraged to tap into the causal relationship between metacognition and creativity through experimental manipulation. Additionally, it should be noted that the majority of participants in this study were female, even though previous research found no gender differences in creative performance (Abraham 2016). Future research is encouraged to increase the sample size and balance male and female subjects to control for gender and ensure the robustness of the research findings.

One more regrettable aspect is that we did not conduct tests on the participants’ subjective experience of metacognition. This may lead to a lack of complete alignment between the results we measured and the participants’ perception, potentially influencing the outcomes to some extent. It is hoped that future research will focus on this issue to compensate for this regret and ensure the accuracy of the data.

In addition, compared to the original RAT test, the Chinese Compound Remote Associates Test (CCRAT) has altered the nature of its measured associations. Both tests have significant limitations in terms of cultural and language (over) reliance. While previous literature has demonstrated the successful use of CCRAT among Chinese participants, it is still risky to convert the traditional Chinese characters to simplified Chinese characters in this study. Additionally, there are concerns regarding the use of simplified Chinese CCRAT materials in conjunction with eye tracking. Existing research indicates significant differences between reading Chinese characters and alphabetic letters (Tang and Chan 2022; Yu et al. 2021; Zhang and Lauer 2015). Overall, reading Chinese characters involves distinct processing strategies, which influence eye behavior and necessitate consideration of the unique aspects of Chinese character recognition development. Future research should take into consideration and optimize the limitations posed by cultural differences and material presentation in technological applications.

Finally, this study was exploratory in nature and did not consider other relevant variables that may impact the relationship between metacognition and creativity. Future research could explore whether some factors, such as motivation (Urban et al. 2021), knowledge, and experience (Urban and Urban 2023) moderate or mediate the relationship, which is helpful to improve our understanding of the mechanisms underlying the relationship between metacognition and creativity.

## 7. Conclusions

Individuals with high metacognitive ability are better at creative thinking, especially in terms of divergent thinking. In addition, they exhibit greater fixation and saccade count, as well as smaller average saccade amplitude on task stimuli, reflecting that they are better at focusing attention on key information and monitoring and regulating cognition in the top-down manner. The documented findings provide the basis for cultivating creativity by enhancing individuals’ metacognitive ability.

## Data Availability

Not applicable.
